# MERRILL: Micromagnetic Earth Related Robust Interpreted Language Laboratory

**DOI:** 10.1002/2017GC007279

**Published:** 2018-04-06

**Authors:** Pádraig Ó Conbhuí, Wyn Williams, Karl Fabian, Phil Ridley, Lesleis Nagy, Adrian R. Muxworthy

**Affiliations:** ^1^ School of GeoSciences University of Edinburgh Edinburgh United Kingdom; ^2^ Geological Survey of Norway Trondheim Norway; ^3^ Department of Geology University of Tromsø Tromsø Norway; ^4^ Cray UK Ltd, Broad Quay House, Broad Quay Bristol United Kingdom; ^5^ Natural Magnetism Group, Department of Earth Science and Engineering Imperial College London London United Kingdom

**Keywords:** micromagnetism, micromagnetic modeling, rock magnetism, mineral magnetism, paleomagnetism

## Abstract

Complex magnetic domain structures and the energy barriers between them are responsible for pseudo‐single‐domain phenomena in rock magnetism and contribute significantly to the magnetic remanence of paleomagnetic samples. This article introduces MERRILL, an open source software package for three‐dimensional micromagnetics optimized and designed for the calculation of such complex structures. MERRILL has a simple scripting user interface that requires little computational knowledge to use but provides research strength algorithms to model complex, inhomogeneous domain structures in magnetic materials. It uses a finite element/boundary element numerical method, optimally suited for calculating magnetization structures of local energy minima (LEM) in irregular grain geometries that are of interest to the rock and paleomagnetic community. MERRILL is able to simulate the magnetic characteristics of LEM states in both single grains, and small assemblies of interacting grains, including saddle‐point paths between nearby LEMs. Here the numerical model is briefly described, and an overview of the scripting language and available commands is provided. The open source nature of the code encourages future development of the model by the scientific community.

## Key Points

MERRILL is a powerful micromagnetic modeling package for the Earth Sciences community which uses a simple scripting language interface
MERRILL can model phenomena in magnetic minerals, like remanence and hysteresis, without need for specialist computational knowledge
This article is an introduction to micromagnetic modeling and MERRILL, including example scripts useful for rock‐magnetism and paleomagnetism

## 1 Introduction

Paleomagnetic observations have contributed a wealth of information about the evolution of the Earth and other planetary bodies (Dunlop & Özdemir, 2001; Merrill, 1998), through the interpretation of remanent magnetization in naturally occurring magnetic minerals. The vast majority of the natural magnetic archives are recorded by nanosized particles whose magnetic properties initially could only be inferred from experimental observations, either on natural samples or on man‐made analogues of bulk particle arrays which have relatively broad particle size distributions. Rock magnetic interpretation is typically based on the assumption that paleomagnetic samples are dominated by single‐domain particles which are theoretically accessible by Néel's theoretical description (Néel, 1955).

With the advent of numerical micromagnetics and high‐resolution imaging techniques, it has been demonstrated that many of the remanence carriers in natural samples are inhomogeneously magnetized pseudo‐single‐domain (PSD) grains, which in their simplest form take on a single vortex (SV) magnetic structure. The first three‐dimensional micromagnetic models were introduced in the late 1980s (Schabes & Bertram, 1988; Williams & Dunlop, 1989), and over the last 30 years have significantly advanced our understanding of magnetic recording in interacting and noninteracting nonuniformly magnetized particles. In paleomagnetism, for example, such studies have demonstrated not only that small PSD grains, ubiquitous in rocks, primarily occupy SV states but that these states can provide reliable and stable recording of the ancient magnetic field that remain stable of over billions of years (Nagy et al., 2017).

Modern desktop computers and workstations are now powerful enough to perform micromagnetic simulations of a wide range of grain sizes, materials, and even clusters of grains that are of interest to rock‐magnetists, paleomagnetists, and environmental magnetists. The currently available open source programs (e.g. OOMMF (Donahue & Porter, 1999), Magpar (Scholz et al., 2003), and NMag (Fischbacher et al., 2007)) are developed for applications in material science and physics and require substantial specialist knowledge to install, maintain, and apply in Earth related contexts. The objective of this article is to introduce an open source micromagnetic model that we believe can significantly increase the ability of the rock and paleomagnetic community to define the magnetic properties of their samples through forward modeling of the behavior of their possible domain states.

We describe here a finite element micromagnetic modeling package called MERRILL (Micromagnetic Earth Related Robust Interpreted Language Laboratory) in honor of the early work of Ron T. Merrill on micromagnetics in rock magnetism (Merrill, 1977; Moon & Merrill, 1984, 1985). MERRILL is a script based modeling program designed for the Earth science community that needs no specialist computing knowledge or proprietary additional software to run the models and visualize the magnetic domain structures. Yet it is a fully tested research‐strength modeling platform that uniquely provides specific features relevant for natural samples and may even outperform some of the above mentioned software in certain applications. Both, precompiled binaries and the FORTRAN source code are freely available and run on LINUX, macOS, and Windows. They can be downloaded from the MERRILL homepage at http://www.rockmag.org.

### 1.1 Workflow

**Figure 1:**
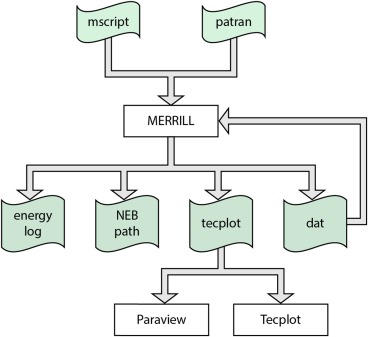
A typical workflow using MERRILL.

A typical workflow using MERRILL is outlined in Figure 1. A tetrahedral mesh of the geometry of interest must first be generated in an external program (although MERRILL contains routines for generating some commonly used geometries), representing the magnetic material. An “MScript” command file is then passed to MERRILL to drive the model, e.g., loading the mesh, setting material parameters, varying external fields, minimizing the micromagnetic energy, and outputting the magnetization to disk. When the magnetization has been output to disk, an external visualization program can be used to inspect the results. The visualizations of the geometries and magnetizations presented in this paper were generated with ParaView (Ahrens et al., 2005). This magnetization may also be used as a starting point in future models.

A productive workflow might involve running a micromagnetic model, inspecting the results visually, and then continuing the model using the previous results as the new starting point, until some desired result is achieved.

## 2 Micromagnetism

In micromagnetism, a real physical magnetic system is described in terms of a continuous vector valued function:

$$\vec{M}:\;{\mathbb{R}}^{3}\;\to \;{\mathbb{R}}^{3},$$

$$\vec{x}\;\;\;\mapsto \;\;\;\vec{M}(\vec{x}),$$

where $\vec{M}(\vec{x})$ represents a mathematical magnetization vector at the mathematical point $\vec{x}\in {\mathbb{R}}^{3}$ (Brown, 1963; Hubert & Schäfer, 1998). All physical energies and processes are then mathematically studied through this continuous function. To link the results to the physical situation, one can assume that the magnetization at a given mathematical point is constructed by averaging the discrete physical sources of magnetism, e.g., electron spins and orbitals, over a small volume centered at that point. This volume must be large enough that the behavior of individual atoms are averaged out, but small enough to resolve inhomogeneous magnetic structures such as domain walls, vortices, or flower states. It turns out that this continuum approximation represents real magnetization structures astonishingly well, even if the mathematical magnetization at a given point represents only the average over a few atoms. Quantum mechanical effects, which are essential in magnetism, are represented purely phenomenologically through material constants like the exchange constant or the magnetocrystalline anisotropy constant.

In micromagnetic models, the magnetic structure in a particle gives rise to various contributions to the total free magnetic energy functional $E(\vec{M})$, or to their associated effective fields. The effective field $\vec{H}$ is given as a function of $\vec{M}$ as

(1) $$H_{i}^{\text{eff}}(\vec{M})=-\frac{\partial E}{\partial M_{i}}(\vec{M}).$$

The dynamics of a micromagnetic system is described by the Landau‐Lifshitz‐Gilbert (LLG) equation (Gilbert, 2004):

(2) $$\frac{\partial M}{\partial t}=-\gamma \vec{M}\times {\vec{H}}^{\text{eff}}(\vec{M})-\lambda \vec{M}\times (\vec{M}\times {\vec{H}}^{\text{eff}}(\vec{M})),$$

where *γ* is the electron gyromagnetic ratio and *λ* is a material‐dependent damping factor. This behaves like a dynamical system where a force ${\vec{H}}^{\text{eff}}$ acts on a system with a nonzero angular momentum vector pointing parallel to $\vec{M}$. A sufficient condition for an equilibrium magnetization ${\vec{M}}^{0}$ is given by

(3) $${\vec{M}}^{0}\times {\vec{H}}^{\text{eff}}({\vec{M}}^{0})=0.$$

This solution is local energy minimum (LEM), which we might associate with a remanent magnetization state, and so we will denote it ${\vec{M}}^{\text{rem}}$. From equation (3) we can see two types of solutions. Either ${\vec{M}}^{0}$ is parallel to ${\vec{H}}^{\text{eff}}({\vec{M}}^{0})$ or ${\vec{H}}^{\text{eff}}({\vec{M}}^{0})=0$. In MERRILL, we focus on the second solution, that is, we solve for the LEM solutions by optimizing the expression for the total free magnetic energy of the system. This is generally much more efficient at finding stable domain structures that the full solution to the LLG equation, and we are not normally interested in the details of the domain transition dynamics that the LLG describes.

### 2.1 Effective Fields

The total effective field ${\vec{H}}^{\text{eff}}(\vec{M})$ for a typical cubic ferromagnetic crystal involves four primary components: Zeeman, anisotropy, exchange and demagnetizing fields (Brown, 1963; Kittel, 1949).

#### 2.1.1 Zeeman Field

The Zeeman field represents the interaction of external sources of magnetic field with the magnetic system under investigation. As such, it is often referred to as the “external field.” It is assumed that the system under investigation has no effect upon the external source.

(4) $${\vec{H}}^{\text{zm}}=\text{constant}.$$

#### 2.1.2 Anisotropy Field

The anisotropy field couples the magnetization to the crystal lattice. It is the primary mechanism by which the symmetries of the lattice affect the magnetization. For a cubic ferromagnetic crystal, if the crystal axes are along the *x*, *y*, and *z* coordinate axes, it can be written

(5) $${\vec{H}}^{\text{anis}}=2K_{1}\left(\alpha_{1}(\alpha_{2}^{2}+\alpha_{3}^{2})\;,\;\alpha_{2}(\alpha_{3}^{2}+\alpha_{1}^{2})\;,\;\alpha_{3}(\alpha_{1}^{2}+\alpha_{2}^{2})\right)$$

with *K*_1_ the anisotropy constant. In magnetite and iron higher‐order terms are much smaller and can be safely ignored for micromagnetic calculations. However, MERRILL does allow a value for *K*_2_ to be set.

The vector $\vec{\alpha }$ here represents the directional cosines of the magnetization with respect to the crystal axes. For a crystal with cubic axes $\vec{a},\;\vec{b}$, and $\vec{c}$, the vector $\vec{\alpha }$ is defined:

(6) $$\vec{\alpha }=\left(\frac{\vec{M}\cdot \vec{a}}{\left|\vec{M}||\vec{a}\right|}\;,\;\frac{\vec{M}\cdot \vec{b}}{\left|\vec{M}||\vec{b}\right|}\;,\;\frac{\vec{M}\cdot \vec{c}}{\left|\vec{M}||\vec{c}\right|}\right),$$

such that $\vec{\alpha }\cdot \vec{\alpha }=1$.

For other noncubic anisotropies, appropriate equations are used, e.g., for a crystal with a uniaxial symmetry, e.g., a tetragonal mineral like tetrataenite, a uniaxial anisotropy ${\vec{H}}^{\text{anis}}(\vec{M})=K_{1}{\left(\vec{M}\cdot \vec{a}\right)}^{2}$ is commonly used, with *a* the anisotropy axis.

#### 2.1.3 Exchange Field

The exchange field serves to align nearest neighbor magnetizations. Although this incorporates quantum mechanical spin coupling, in the continuum approximation a micromagnetic expression can be given as

(7) $${\vec{H}}^{\text{exch}}=A\nabla^{2}\vec{\alpha }$$

with *A* the exchange coupling constant.

#### 2.1.4 Demagnetizing Field

The demagnetizing field represents the magnetic field generated by the magnetic material itself, derived from the “magnetic self‐energy.” It is called the demagnetizing field, because a higher magnetic self‐energy represents a higher energy configuration, so it typically acts on the magnetization in such a way that it minimizes external flux and thereby itself.

(8) $${\vec{H}}^{\text{dmag}}\;=\;\vec{\nabla }\phi ,$$

(9) $$\nabla^{2}\phi \;=\;\vec{\nabla }\cdot \vec{M},$$

(10) ϕ(∞) = 0.

Outside the magnetic material, this is the “stray field.”

## 3 Minimum Energy Solutions Using Finite Elements

There are a variety of numerical micromagnetic approaches than can be used to solve for locally stable magnetic domain structures. Here we use a method that is both numerically efficient and robust while requiring the simplest input for representing the geometry of the grain. In most micromagnetic models, the primary consideration is the efficiency with which in the internal demagnetizing field can be computed. This field calculation scales as $O(N^{2})$, where *N* is the number nodes, but if the geometry is meshed using a regular grid this scaling can be reduced to O(Nlog⁡N) using FFT (Fabian et al., 1996; Wright et al., 1997). However, such regular grids do not easily account for arbitrary grain geometries, although some success has been achieved by relative scaling of surface elements (Witt et al., 2005), or irregular FFT techniques (Kritsikis et al., 2008).

In MERRILL, we employ the Finite Element Method (FEM), a standard technique for describing functions over a geometry and solving differential equations in terms of those functions (Davies, 2011). MERRILL uses arbitrarily shaped linear tetrahedral finite elements to describe the geometry of a particle and to solve for the LEM stable domain states. Some care is needed in the calculation of the demagnetizing field described by equations (8), (9), (10), which involves solving the Poisson equation for the magnetic scalar potential over an infinite space.

MERRILL makes use of a Boundary Element Method (BEM) technique (Fredkin & Koehler, 1990; Lindholm, 1984), which is a specialization of the FEM for homogeneous Poisson equations, particularly suited to problems defined over an infinite space. This method has significant advantage in that we need not create a mesh in the free‐space region outside the geometry of the magnetic particle (even for multiparticle solutions) but the method does, however, increase the memory requirements of the programme. In our experience, this is acceptable for single‐grain geometries requiring up to about a million elements. A more detailed account of different mciromagnetic methodologies can be found in Fidler and Schrefl (2000).

LEM states are found by solving for the minimum free magnetic energy of the system $E=-{\vec{H}}^{\text{eff}}\cdot \vec{M}$ where the total effective field is given by

(11) $$\begin{array}{l} {\vec{H}}^{\text{eff}}(\vec{M})\;={\vec{H}}^{\text{exch}}(\vec{M})+{\vec{H}}^{\text{anis}}(\vec{M})\\ +\;{\vec{H}}^{\text{zm}}(\vec{M})+{\vec{H}}^{\text{dmag}}(\vec{M}). \end{array}$$

To include further phenomena, like stress fields or surface anisotropy, in theory one need only derive the corresponding effective field, typically by taking the derivative of the energy with respect to the magnetization, and add it to equation (11).

In order to determine minimum energy solutions, by default MERRILL makes use of an accelerated adaptive step‐size steepest descent algorithm across the energy landscape, optimized for micromagnetics, here called “Hubert Minimizer” (Berkov, 1998a; Ramstöck, 1997). A standard conjugate gradient optimizer is also available as an option, but in most cases is slightly less efficient in finding energy minima.

## 4 Mesh Generation

MERRILL requires that the geometry of the magnetic particle is described using a linear tetrahedral mesh (by default defined in micron units). The magnetization is thus specified only at the four vertices of each element and linearly interpolated at all other locations. MERRILL is able to generate suitable meshes for simple grain geometries such as cubes and spheres, however, more complex particle geometries require additional meshing software of which there are many free and commercial programmes. The only requirement is that the mesh is formatted according to ASCII text based PATRAN (.neu) standard. Most finite element meshing applications will support this and more detailed information about the PATRAN format can be obtained from the website of the MSC Software Corporation (https://simcompanion.mscsoftware.com).

Like all finite element models, the quality of the mesh will affect the convergence efficiency of the model. In most cases meshing software will take care to produce a qood quality mesh, but for highly irregular geometries problems may still arise. Discussion of mesh quality metrics can be found in many publications (e.g., Dai et al., 2014; Knupp, 2006)

For micromagnetic applications, it is important to ensure that that mesh is fine enough to resolve the expected spatial variation of the magnetization within the model geometry. The maximum element size is usually described in terms of the “exchange length,” $l_{\text{exch}}$ (Rave et al., 1998), which is dependent on the magnetic material parameters:

(12) $$l_{\text{exch}}=\sqrt{\frac{2A}{\mu_{0}M_{s}^{2}}}.$$

For iron and magnetite, for example, the exchange length is around 3 and 9 nm, respectively, at 30°C and slightly larger near the Curie temperature.

Clearly, the bigger particle and finer the mesh the longer the model will take to converge to a LEM state. For large grains, the mesh size is usually set to the exchange length. However, small grain geometries will require a mesh that is finer than the exchange length in order adequately represent the grain shape.

## 5 Model Validation

Validation of micromagnetic models should ultimately be done against experimental observations. However, such direct validation has until recently been extremely difficult to achieve since the maximum grain size that could be modeled numerically was much smaller than that which could be directly observed experimentally. As a result, the earliest micromagnetic models could only be validated against bulk observations such as assemblies of sized particle fractions or magnetosome observations (Fabian et al., 1996; Williams & Dunlop, 1995; Witt et al., 2005) in the case of natural materials, or on thin film and particulate man‐made recording media (Labrune & Miltat, 1990; Silva & Bertram, 1990). However, results from MERRILL have been directly compared with nanoscale experimental data via electron holography with good agreement (Almeida et al., 2016).

With an increasing number of micromagnetic models being published, a number of standard tests were developed in order to ensure that the models were at least self‐consistent. Validation against these standard tests is now a prerequisite for any newly published micromagnetic code. One such test is *μ*MAG Standard Problem 3 (see Appendix A), which tests for the critical edge length of a cube for transition between a flower state and a vortex state. Our solutions found the flower and vortex states had equal energies at an edge length of 8.47 $l_{\text{exch}}$ when extrapolated to an infinitely fine mesh, which is in good agreement with other submissions to the *μ*MAG problem.

MERRILL also tests the effective field components against some analytic solutions. For example, the demagnetizing field for a uniform sphere can be written ${\vec{H}}^{\text{dmag}}=\frac{1}{3}\vec{M}$ and should be independent of the sphere size.

## 6 MScript: The MERRILL Scripting Language

MERRILL is run at the command line with an input text file containing a series of MScript commands for MERRILL to execute. These commands allow the user to interact with MERRILL in a number of ways, for example, setting material constants, loading meshes or finding a local energy minimum. In this section, we will outline a few typical computer experiments that can be used to probe the behavior of the magnetization of magnetic materials using MERRILL. This will also serve as a brief tutorial on the MScript language and introduce some of features in MERRILL.

The default units for MERRILL are microns and degrees Celsius, although different units may be used as specified in the user documentation. A full list of MERRILL commands can be found in Appendix B and on MERRILL download page (http://www.rockmag.org) where it will be updated as MERRILL is developed further.

Some typical commands include (but are not limited to)

***Magnetite*** 〈 ***temperature*** 〉 ***C***

Set material constants to magnetite at 〈 *temperature* 〉 degrees Celsius.

***Iron*** 〈 ***temperature*** 〉 ***C***

Set material constants to iron at 〈 *temperature* 〉 degrees Celsius.

***GenerateCubeMesh*** 〈 ***width*** 〉 〈 ***edgelen*** 〉

Generate a cubic geometry of width 〈 *width* 〉 and with the average length between mesh nodes of length 〈 *edgelen* 〉.

***Uniform Magnetization*** 〈x,y,z〉

Set the magnetization of the material to

$$\vec{M}=M_{s}\cdot (x,y,z)/\sqrt{x^{2}+y^{2}+z^{2}}$$

***Minimize***

Run the minimizer to find the local energy minimum.

***WriteMagnetization*** 〈 ***filename*** 〉

Write the magnetization to disk. Two files are written, 〈 *filename* 〉.dat and 〈 *filename* 〉_mult.tec. The file 〈 *filename* 〉.dat contains a list of vertex points and the magnetization at that point. This is suitable for use with the *ReadMagnetization* command (see below). The 〈 *filename* 〉_mult.tec file is the mesh and the magnetization in a TecPlot file format. This can be read by a number of visualization tools, including the free, open source and cross‐platform ParaView software (Ahrens et al., 2005).

***ReadMagnetization*** 〈 ***filename*** 〉

Read a previously written file 〈 *filename* 〉 from the *WriteMagnetization* command, and set the current magnetization based on that.

***External Field Direction*** 〈x,y,z〉

Set the Zeeman field direction to

$${\hat{H}}^{\text{zm}}=(x,y,z)/\sqrt{x^{2}+y^{2}+z^{2}}$$

***External Field Strength*** 〈 ***magnitude*** 〉 ***mT***

Set the Zeeman field strength to

$$|H^{\text{zm}}|=〈magnitude〉$$

MScript also includes supports for variables and loops. For example, the script

**Loop** myvalue 0 100 10

**Print** #myvalue

**EndLoop**

creates a loop with the local variable myvalue. The numbers in the loop command determine initial value, end value and step size, such that the above loop prints out the values 0, 10, 20, 30, 40, 50, 60, 70, 80, 90, and 100. To distinguish between floating point, integer and string values, the variable *myvalue* can be referred to as %myvalue, #myvalue, and $myvalue$, depending on what is needed. For example, in

**Loop** myvalue 0 100 10

**WriteMagnetization** SolnFileA_%myvalue

**WriteMagnetization** SolnFileB_#myvalue

**EndLoop**

the filenames written to by the two **WriteMagnetization** commands are quite different. The argument SolnFileA_%myvalue produces filenames of the form SolnFileA_+0.10000E2 when myvalue is 10, while the argument SolnFileB_#myvalue produces filename of the form SolnFileB_10.

### 6.1 Minimization

A basic minimization from a random start, or a uniformly magnetized start can be a quick way to tell if a grain is in the SD or PSD size range. Moreover, it is the basic building block from which most other MERRILL modeling experiments come.

A simple minimization script to determine a LEM state from a random initial state for a meshed geometry can be given as follows:

*! Setup material constants for Iron*

*! at 20 Degrees Celsius*

**Iron** 20 C

*! Load the meshed geometry from a*

*! Patran Neutral file*

**ReadMesh** 1 mesh.neu

*! Randomize the magnetization*

**Randomize All Moments**

*! Run the minimizer*

**Minimize**

*! Output the solution M to two files*:

*! soln.dat and soln_mult.tec*.

*! The soln.dat file can be used as the*

*! input for another run as, e.g., an*

*! initial guess. soln_mult.tec is a*

*! TecPlot format file which can be used*

*! to view the solution in a visualization*

*! program such as ParaView*

*! (which is free and open source!)*

**WriteMagnetization** soln

**Figure 2:**
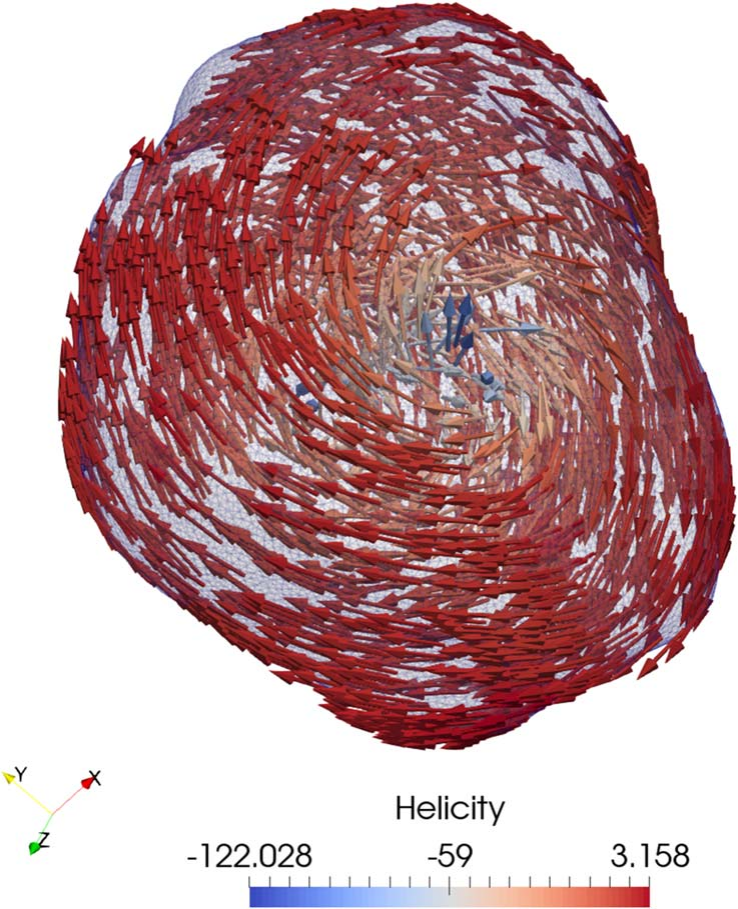
Minimization of an iron inclusion from a dusty olivine sample at 20 C with random initial magnetization. The shape is approximately a prolate ellipsoid with semimajor axes of 0.134 and 0.126 μm, and semiminor axis of 0.085 μm, with axes aligned along the cardinal directions. The LEM is a single vortex state with core aligned along the semiminor axis.

The output of this script can be seen in Figure 2 using a mesh generated from experimental focused ion beam nanotomography data for an example iron inclusion in dusty olivine in a chondritic meteor sample (Einsle et al., 2016).

It is important to note that there may be several possible magnetization states the minimization might settle upon, representing multiple possible, and completely valid local energy minima states. However, from a given starting point, barring numerical noise, the minimization should always reach the same end point.

The LEM found can be highly dependent on the choice of initial state. A simple minimization script to determine a LEM state for a cubic grain of magnetite, starting with uniform magnetization, can be given as follows:

*! Setup material constants for Magnetite*

*! at 20 Degrees Celsius*

**Magnetite** 20 C

*! Generate a.08 micron cube using the*

*! built‐in cube mesh with mesh size 5 ∼nm*

*! generator*

**GenerateCubeMesh** 0.080 0.005

*! A flower state will nucleate from a*

*! uniform magnetization along the*

*! easy axis*

**Uniform Magnetization** 1 1 1

**Minimize**

**WriteMagnetization** flower_soln

**Figure 3:**
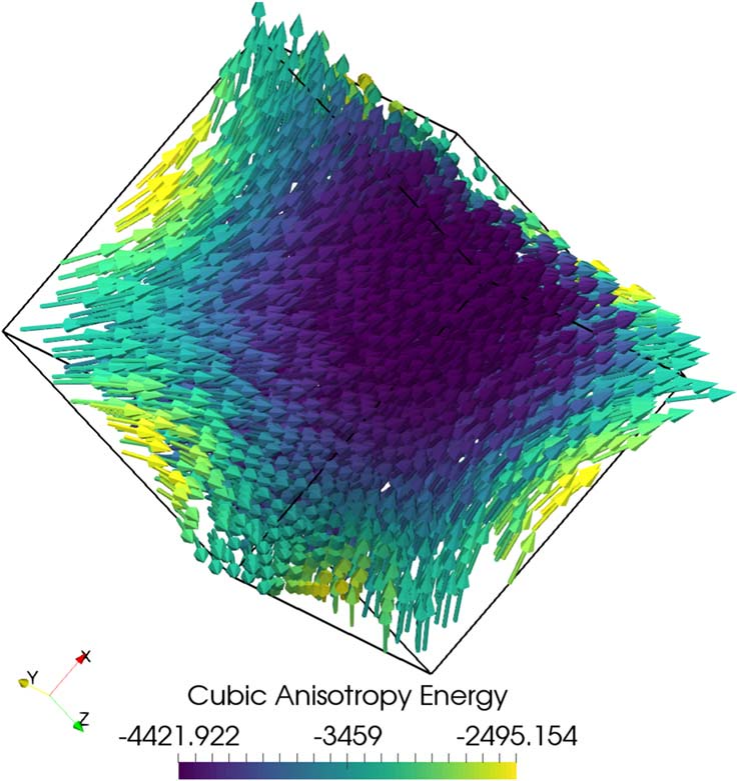
Minimization of a 0.08 μm cube of Magnetite to a flower state from an initial uniform [111] magnetization.

The output of this script can be seen in Figure 3, representing a flower state.

By starting from a rough vortex state rather than a uniform state, we can instead nucleate a vortex LEM. This can be accomplished by

**Magnetite** 20 C

**GenerateCubeMesh** 0.080 0.005

*! A vortex state will nucleate from a*

*! rough vortex state with core aligned*

*! along the easy axis*

*! The 1 1 1 refers to the direction,*

*! 0.02 is the “tightness,” which should*

*! by manually tuned by the user, and*

*! LH will produce a left hand vortex*.

**Vortex Magnetization** 1 1 1 0.02 LH

**Minimize**

**WriteMagnetization** vortex_soln

**Figure 4:**
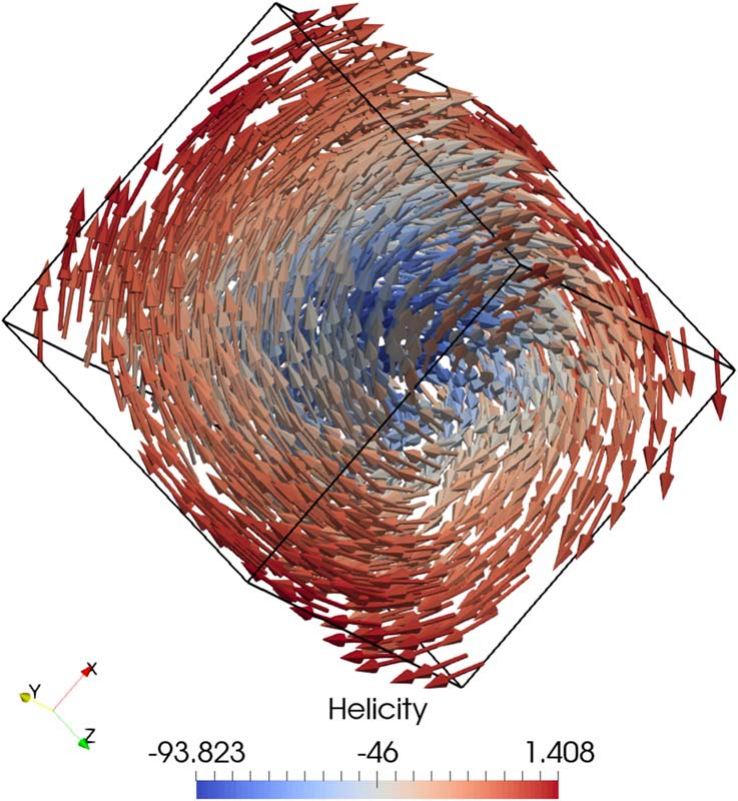
Minimization of a 0.08 μm cube of Magnetite to a vortex state from an initial approximate [111] aligned vortex state, colored by helicity ( $\vec{M}\cdot (\vec{\nabla }\times \vec{M})$).

The output of this new script (seen in Figure 4) highlights the “local” aspect of a local energy minimum.

### 6.2 Hysteresis Loops

Hysteresis loops are a useful tool when a magnetic material has several remanent magnetization states for a given set of parameters. From a hysteresis loop, it is possible to deterministically move from one state to another, and find the tipping point where the variation of a given parameter will cause one state to spontaneously switch to the other. This also provides information on the range of values for the given parameter where both states can coexist. In other words, it provides some information about the stability of a given state when multiple valid states exist.

Since the minimization should always reach the same solution when given the same starting point, a hysteresis loop run with the same parameters and the same changes in parameters should always return the same results in MERRILL.

#### 6.2.1 Magnetic Field Hysteresis

A magnetic hysteresis loop has several uses. A single hysteresis loop can be useful to get a feel for the behavior of the magnetization of a system (i.e., its coercivity, if it is SD or PSD). The average of hysteresis loops in many directions can be used to compare simulations with experimental observations of magnetic characteristics such as coercivity.

A hysteresis loop is a quasi‐static thermodynamic process. That is to say, at each point of the hysteresis loop, the system is assumed to be in equilibrium and the energy at a local energy minimum. A change from one point to the next in a hysteresis loop represents the system moving from what was a local energy minimum in the previous step to the nearest, new local energy minimum in the current step.

The simulation of a hysteresis loop is accomplished by the following scheme:

1. Set Zeeman field to saturating value in a fixed direction: Z = Zmax.
2. Find LEM.
3. Update Zeeman field: Z = Z − a*Zmax.
4. If Z != −Zmax, go to 2.

For a saturating field ${\vec{Z}}^{\text{max}}$, a hysteresis loop will run from ${\vec{Z}}^{\text{max}}$ to − ${\vec{Z}}^{\text{max}}$ in small increments. Here “small” means sufficiently small enough that any phenomenon sensitive to the external field, e.g., magnetization switching or nucleation of vortices, are accounted for. In practice, “small” means just small enough to resolve changes in the magnetization you are looking for, but as large as possible to reduce the total number of steps needed for the loop. Otherwise a large amount of work may be done unnecessarily for little progress.

A MERRILL script which accomplishes this is

**Magnetite** 20 C

**GenerateCubeMesh** 0.100 0.005

**External Field Direction** 1 0 0

**External Field Strength** 0 mT

**Loop** field −100 100 5

**Randomize Magnetization** 10

**External Field** Strength

**Minimize**

*! Write current value to disk*,

*! so we can inspect it later*.

**WriteHyst** hyst_soln

**EndLoop**

Since hysteresis is generally symmetrical to forward and reverse fields, it is only necessary to run either the upper or lower branch of the hysteresis loop, not both.

**Figure 5:**
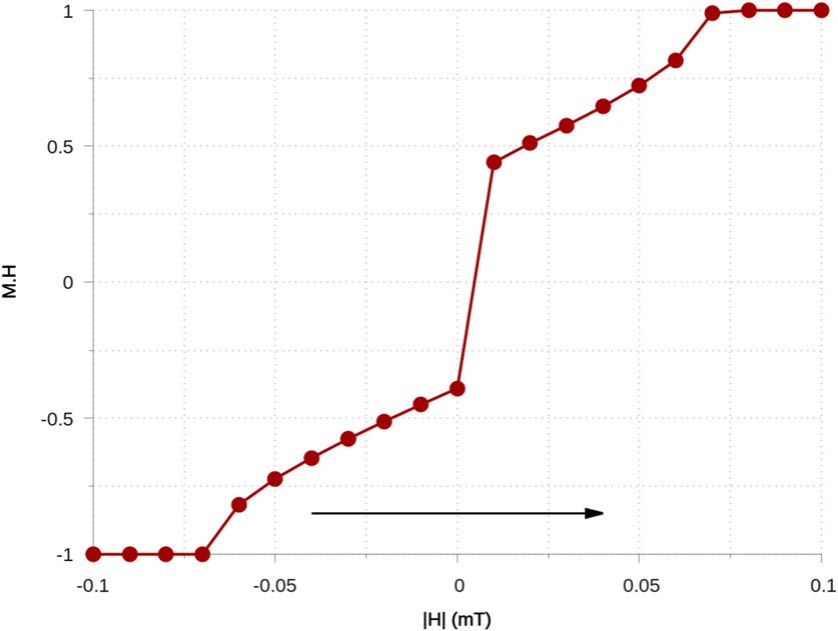
The lower branch of a hysteresis loop with respect to an external magnetic field, varying from −100 to 100 mT for a 0.1 μm sphere of magnetite. The arrow denotes the direction of change of |H| during the loop.

**Figure 6:**
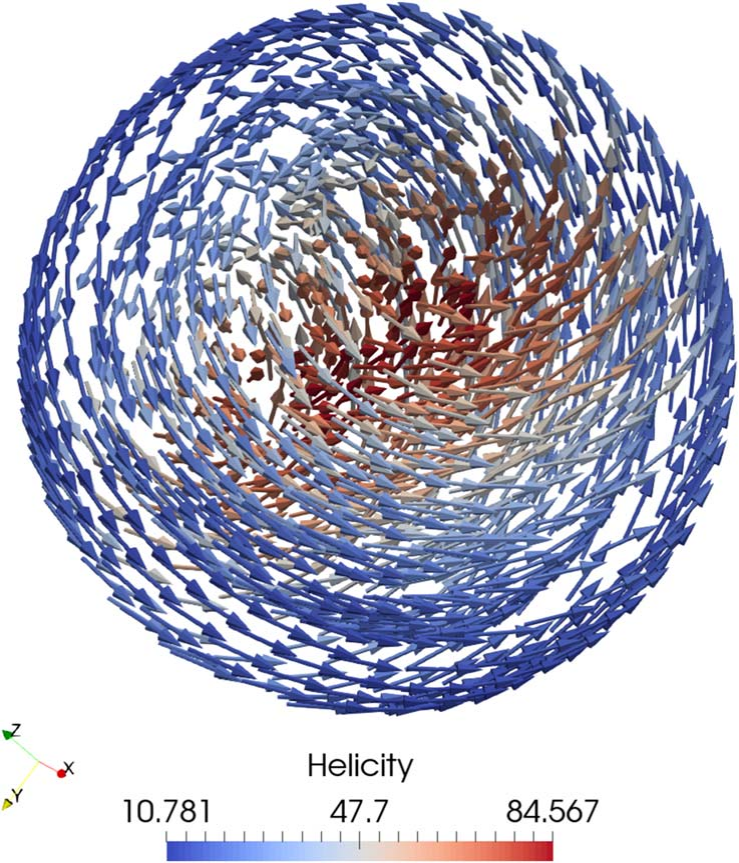
Magnetization of a 0.1 μm sphere of magnetite at |H|=−30 mT during a field hysteresis from |H|=−100 mT to *H* = 100 mT, colored by helicity ( $\vec{M}\cdot (\vec{\nabla }\times \vec{M})$).

A plot of the upper branch is shown in Figure 5. This curve is different to the usual SD curve, where the value $\vec{M}\cdot \vec{H}$ remains near the saturation value, until passing the coercive field. Here the grain enters a vortex state, and the value $\vec{M}\cdot \vec{H}$ can minimized while keeping the anisotropy energy relatively low by varying the shape of the vortex. An example of the vortex state at |H|=−30 mT is shown in Figure 6.

#### 6.2.2 Size Hysteresis

A hysteresis loop where the size of the grain is varied rather than an external field can be a useful tool for determining SD and PSD ranges, and the evolution of magnetic domain states. Since several remanent states can exist for a grain, particularly in the early PSD size range, it can be difficult to pinpoint exactly where that regime begins.

In a size hysteresis loop, the grain is started in an SD magnetization state. The size of the grain is increased until it spontaneously switches to a vortex PSD state. The size of the grain is then scaled down until it is in a SD state again. The branch of increasing size can tell us what the largest grain size is that supports an SD state, and the branch of decreasing size can tell us what the smallest grain size is that supports a PSD state.

Note that the exact initial SD was not defined. Ideally, a size hysteresis should be run for each possible SD remanent state. For symmetric grains, symmetry should make many of these redundant. However, for asymmetric grains, the direction of the initial magnetization may greatly affect the stability of the SD solution.

When scaling sizes, it is important to consider the size of the elements of the mesh during scaling. Typically, the edges of a mesh should be around the exchange length. When the size of a mesh is increased, the average edge length should not exceed the exchange length. By starting with a larger mesh and scaling down, rather than the other way around, we can avoid this problem. However, finer meshes take longer to run. A compromise can be found using the “Remesh” command. A user might use a mesh that is suitable for scaling up to, e.g., 0.1 μm, and at that size, switch to a mesh suitable for up to, e.g., 0.2 μm.

So if we want a maximum node spacing of, e.g., 0.005 μm, we make two meshes of width 0.1 μm: one with node spacing 0.005 μm, and one with node spacing of 0.0025 μm. The first mesh will cover grains from 0 to 0.1 μm, while the second can cover grains from 0.1 to 0.2 μm. We make the meshes the same initial size so the Remesh command can interpolate the magnetization directly from one to the other.

In MERRILL, the “Resize” command can be used to scale the mesh. An example size hysteresis script incorporating all of this is

*! Use magnetite material parameters*

**Magnetite** 20 C

*! Ensure we can load at least 2 meshes*

*! at a time*

**Set MaxMeshNumber** 2

*! Load 0.1 um mesh with 0.005 um node*

*! spacing, suitable for scaling up to*

*! 0.1 um, into slot 1*

**ReadMesh** 1 octahedron_0.1um_0.005um.neu

*! Load 0.1 um mesh with 0.0025 um node*

*! spacing, suitable for scaling up to*

*! 0.2 um, into slot 2*

**ReadMesh** 2 octahedron_0.1um_0.0025um.neu

*! Set reference size of the meshes to 100*.

*! We'll be scaling from 10 to 200*.

*! In this case, 100 will be 0.1 um*,

*! 10 will be 0.01 um, and 200 will be*

*! 0.2 um*.

**define** refsize 100

*! Make sure mesh 1 is loaded*

**LoadMesh** 1

*! Set initial magnetization to [111]*

**Uniform Magnetization** 1 1 1

*! Loop from 10 to 100 in steps of 10 for*

*! the 0.005 um mesh*

**Loop** meshsize 10 100 10

*! Resize our mesh to the current*

*! %meshsize*

*! For #meshsize 20, for example*,

*! the 0.1 um mesh is scaled to*

*! 0.02 um*.

**Resize** #refsize #meshsize

*! Give the magnetization a small kick*

**Randomize Magnetization** 5

! Run the minimization

**Minimize**

*! Write the output to a file*

**WriteMagnetization** up_0.$meshsize$um

*! Resize the mesh back to its original*

*! size, for the next loop iteration*

**Resize** #meshsize #refsize

**EndLoop**

*!*

*! Hand off to the 0.0025 um mesh*

*!*

*! Interpolate the current magnetization to*

*! mesh 2 and load it*

**Remesh** 2

*! Loop from 110 to 200 in steps of 10 for*

*! the 0.0025 um mesh*

**Loop** meshsize 110 200 10

**Resize** #refsize #meshsize

**Randomize Magnetization** 5

**Minimize**

**WriteMagnetization** up_0.$meshsize$um

**Resize** #meshsize #refsize

**EndLoop**

*! Small to large done*.

*! Now do large to small*.

*! Loop over 0.0025 um mesh*

**Loop** meshsize 200 110 −10

**Resize** #refsize #meshsize

**Randomize Magnetization** 5

**Minimize**

**WriteMagnetization** down_0.$meshsize$um

**Resize** #meshsize #refsize

EndLoop

*! Hand off to 0.1 um mesh*

**Remesh 1**

*! Loop over 0.1 um mesh*

**Loop** meshsize 100 10 −10

**Resize** #refsize #meshsize

**Randomize Magnetization** 5

**Minimize**

**WriteMagnetization** down_0.$meshsize$um

**Resize** #meshsize #refsize

**EndLoop**

**Figure 7:**
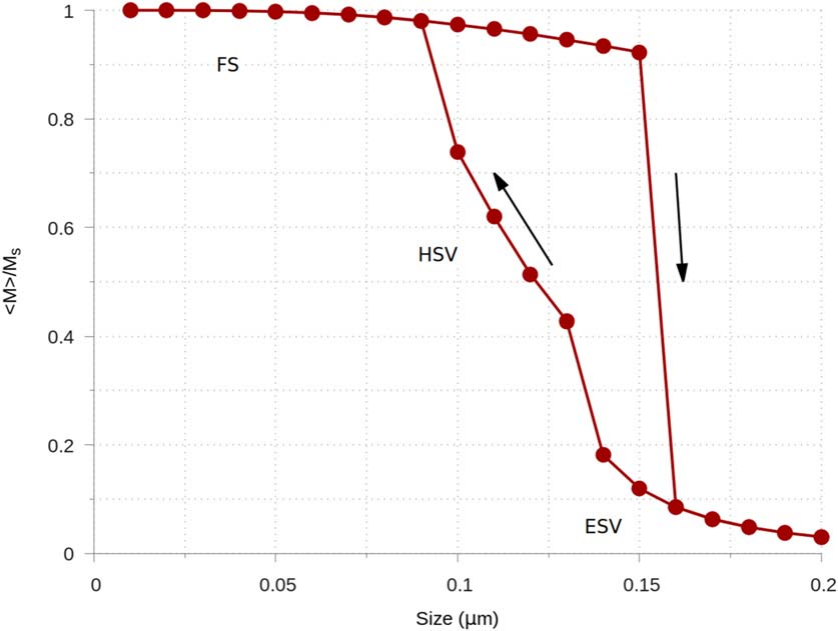
A full hysteresis loop of saturation magnetization versus size for an octahedral grain of magnetite. Three distinct magnetic phases are marked: a flower state (FS), a hard‐aligned single vortex state (HSV), and an easy‐aligned single vortex state (ESV).

**Figure 8:**
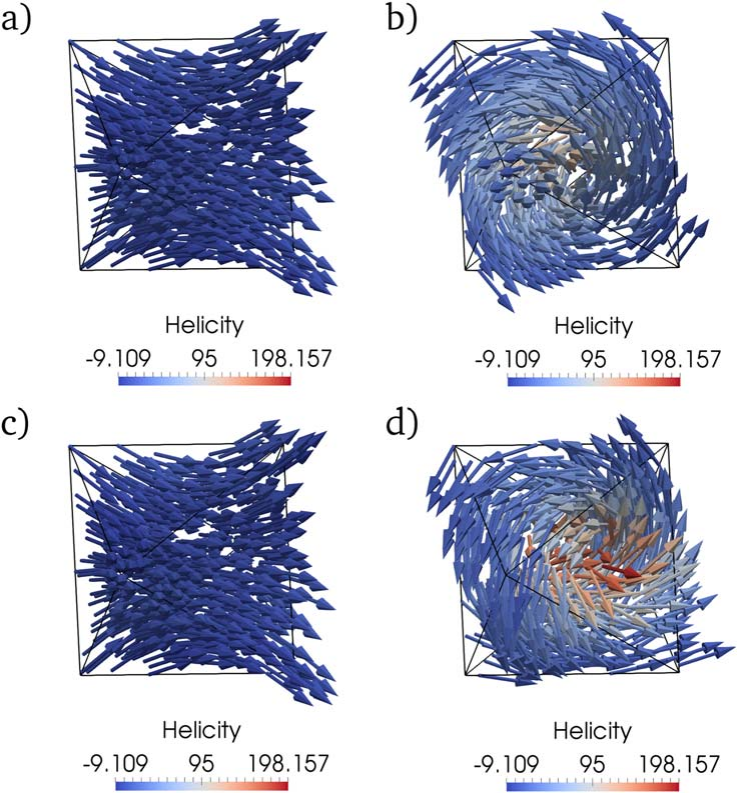
Comparison of upper and lower branch results for a size hysteresis of an octahedral grain of magnetite. The upper branch goes from small to large, and the lower branch goes from large to small. (a) The upper branch at 0.13 μm, (b) the lower branch at 0.13 μm, (c) the upper branch at 0.15 μm, and (d) the lower branch at 0.15 μm. The coloring is helicity ( $\vec{M}\cdot (\vec{\nabla }\times \vec{M})$).

A graph of the saturation magnetization versus the grain size is shown in Figure 7, with three distinct magnetic phases marked: a flower state (FS), a hard‐aligned single vortex (HSV), and an easy‐aligned easy vortex state (ESV). A comparison of the upper and lower branches at 0.13 μm and at 0.15 μm, are shown in Figure 8, demonstrating the ESV and HSV states for the lower branch, and the corresponding FS states on the upper branch.

#### 6.2.3 Temperature Hysteresis

A temperature hysteresis can be used to observe the effects of heating, say iron, from room temperature to the Curie temperature and back. The approach MERRILL takes to this is to simply change the temperature dependant material parameters for each step. In this way, the model is of a “cold” material, since no other thermal effects are taken into account which might spontaneously and nondeterministically effect the magnetization. However, the changes in material properties can have significant impact on the shape of the energy landscape with respect to the depths, positions and even number of the local energy minima.

An example temperature hysteresis of iron is

**GenerateCubeMesh** 0.18 0.005

**Uniform Magnetization** 1 1 1

**Loop** temperature 20 770 10

*! Set the material parameters for iron*

*! at the given temperature*

**Iron** %temperature C

*! Find the LEM*

**Minimize**

*! Write current value to disk, so we*

*! can inspect it later*.

**WriteHyst** hyst_soln_#temperature

**EndLoop**

### 6.3 Energy Barriers

MERRILL includes a new method for finding the minimum energy transition between two given LEM states (K. Fabian & V. P. Shcherbakov, Energy barriers in three‐dimensional micromagnetic models and the physics of thermo‐viscous magnetization in multidomain particles, arXiv 1702.00070v1, 2017). It uses a combination of the Nudged Elastic Band technique (Dittrich et al., 2002; Henkelman & Jónsson, 2000) and an action minimization method (Berkov, 1998a, 1998b; Fabian & Shcherbakov, arXiv 1702.00070v1, 2017). It finally constructs a prescribed number of intermediary states between the given LEM states *L*_1_, *L*_2_, such that their interpolation represents the average physical switching path for a fully damped (*λ* = 0) switching process from *L*_1_ to *L*_2_. The path with the lowest maximum energy barrier determines the thermal activation energy required to switch between these states. From that, the probability of switching and the relaxation time across the energy barrier can be obtained.

These quantities are of paramount importance in paleomagnetism, where small changes in size, shape, or material parameters can change the relaxation time from the order of seconds, to millions of years, and the corresponding paleomagnetic information is either completely unblocked, or can be regarded as stable or blocked. In contrast to, for example, the simulation of writing heads for magnetic hard drives, the nanosecond dynamics of the switching processes are of no interest in paleomagnetism, such that time‐consuming full‐fledged LLG models do not improve the result. On the contrary, the exact determination of the energy barrier through the saddle‐point path provides a better estimate of the thermal relaxation time. Note in that respect that the LLG equation does not include temperature, and that thermal activation has to be added in a phenomenological way, e.g., as a stochastic zero‐mean Gaussian fluctuation field (Torres et al., 2001), if it is considered at all.

If morphology and composition of a natural magnetic mineral system is known MERRILL can provide quantitative insight to whether the signal recorded has remained stable since it was recorded (Nagy et al., 2017).

An example script for a NEB calculation is

*! Use only one mesh for this NEB*

*! minimization*

**Set MaxMeshNumber** 1

*! Read in the mesh*

**ReadMesh** 1 model.neu

*! Set the maximum number of energy*

*! evaluations for LEM/path calculations*

**Set MaxEnergyEvaluations** 10000

**Set MaxPathEvaluations** 1000

*! Set the material parameters using a*

*! predefined material (iron)*

**Iron** 20 C

*! Define an initial path containing only*

*! two points (the start and end points)*

**Set PathN 2**

*! Read in the start structure from a*

*! magnetization file & store as path*

*! point 1*

**ReadMagnetization** start_mag

**MagnetizationToPath** 1

*! Read in the end structure from a*

*! magnetization file & store as path*

*! point 2*

**ReadMagnetization** end_mag

**MagnetizationToPath** 2

*! Define the energy log output file*

**EnergyLog** nebinitial_energy

*! Refine the path to 100 structures (98*

*! intermediate structures are created)*

**RefinePathTo** 100

*! Set the minimization to use conjugate*

*! gradient*

**ConjugateGradient**

*! Set the exchange calculator to the*

*! typical one (default)*

**Set ExchangeCalculator** 1

*! Generate an initial path and save the*

*! initial path*

**MakeInitialPath**

**WriteTecPlotPath** initialpath.tec

*! Run the NEB using the initial and write*

*! the output file*

**PathMinimize**

**WriteTecPlotPath** finalpath.tec

*! Output the energy of each point of the*

*! path to disk*.

**PathStructureEnergies** energies

## 7 Practical Considerations

**Figure 9:**
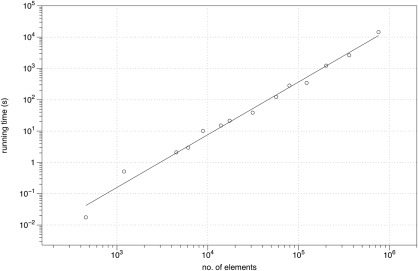
A log‐log plot of the time taken for MERRILL to find a local energy minimum of a cube, starting from a random state, versus the number of elements in the mesh.

There are a number of practical considerations when using MERRILL. Presently, MERRILL is not parallelized. However, many instances of MERRILL may be run in task‐farm parallel manner for reasonably small grains. This approach can be used to model, for example, large assemblies of noninteracting grains, and hysteresis loops about many axes in parallel. The maximum grain size that you will be able to model will be dependent on a number of factors, but primarily the number of elements in the model. MERRILL will comfortably cope with models of up to approximately 10^6^ elements for single grain of simple geometry on a machine with access to 64 GB of RAM (see Figure 9). If the model consists of a cluster particles where the total grain surface to volume ratio is significantly greater than that of a single grain then the maximum number of elements in the model would need to be less than 10^6^. It is not possible to formulate any hard rules that cover the combination of different number of particles, grain sizes and mesh sizes, but the user should be aware that the memory requirement increases as the square of the number of surface nodes (which is typically proportional to the number of surface elements).

The time taken for a model to converge on a local energy minimum will again be largely dependent on the model size (see Figure 9), but also on how close your initial guess is to the final LEM solution. Thus solution times for a random initial guess and a uniform saturated state initial guess can have significantly different solution times. A similar situation occurs during a change in the magnetic phase, i.e., when a solution changes from SD to SV, or hard‐aligned SV to easy‐aligned SV.

In rare cases, the model might attempt a large number of iterations because of slow convergence to the final solution. In such cases it is better to place a limit in on the maximum number of allowed iterations (which has default value of 100,000). Often such slowly converging solutions can be avoided by trying a slightly different initial guess, slightly changing the model mesh, or adding a small random kick to the solution.

As mentioned in section 4, the mesh shape and quality also impact on convergence times. Regular tetrahedra are the most numerically stable elements, so the more regular the tetrahedra in a mesh, the quicker the model will converge. The single most important concern, however, is to avoid slivers. Slivers are elements that are so flat, their volumes approach zero (Alliez et al., 2017). These lead to extreme numerical instability and very slow convergence times, if the model converges at all. Slivers might occur during Delaunay triangulation, as the algorithm is well known to be blind to a class of slivers where the vertices all lie on the same circumcircle. An additional mesh optimization step like Lloyd optimization, or ODT smoothing is often performed during mesh generation to eliminate slivers. Most finite element meshing packages will have algorithms that attempt to detect and avoid such elements, but in all cases when the model fails to converge it is important to check the mesh quality (e.g., using the “Mesh Quality” filter in ParaView).

Also mentioned in section 4 is that mesh edge lengths should be smaller than the exchange length. In most meshing programs, it is unclear what the defined “mesh size” is. In some cases, it is the upper bound of the radius of the circumcircle of an element allowed before the element is decomposed into smaller elements. In some cases, it is the diameter of this circumcircle. For some software, it is the target average edge length of the elements in the mesh. In any case, the ReportEnergy function of MERRILL reports the average edge length of the mesh in nanometres, which is then directly comparable to the exchange length reported by this function in nanometres. Users are encouraged to use these values as a useful and direct report of whether the mesh is fine enough for the given material.

The solvers used in MERRILL can be susceptible to finding unstable equilibria. Gimbal locking, for instance, can occur for field hysteresis loops directly along the hard‐axis of the material. Two simple techniques can be used to avoid this. First, prefer not to perform hysteresis loops directly along the major axes. Add a slight perturbation, i.e., use the [1 1 1.0001] direction instead of [1 1 1]. Another technique is to add a small random “kick” to the magnetization. Typically, moving each magnetization randomly by around 5 or 10 degrees is not so large that it will move the state out of a deep, stable LEM, but it can move it off a local maximum, and also out of a shallow LEM, where the magnetization is around a SD/SV phase transition.

## 8 Discussion

The formulation of a micromagnetic model in an easy to use form can significantly enhance its application in paleomagnetic and rock magnetic investigations. MERRILL presents such a tool that is particularly focused on finding remanent states and studying their stability. The parallels and usefulness to paleomagnetic and rock magnetic studies should be clear. MERRILL has been used in a number of publications and talks using functionality not presented in this paper, e.g., behaviors of assemblages of interacting grains, large models, strongly anisotropic materials and detailed simulations of magnetostrictive effects (e.g., Almeida et al., 2016; Chang et al., 2012; Conbhuí et al., 2016; Einsle et al., 2016; Li et al., 2013; Nagy et al., 2017; Williams et al., 2010). Not all of these, however, used the scripting interface presented here.

The simple scripting language makes it particularly friendly to nontechnical users. The fast and efficient minimization scheme means simple computer experiments can be run quite quickly. The scripts presented here represent the sort of scripts run by the authors day to day. Some effort has been made to make these copy/pasteable, but a curious reader is recommended to look in the “demo” directory of the MERRILL package for more information and examples.

If the scripting language is not up to a particular task, MERRILL can also be used as a library and called from a Fortran program. In addition, a plugin interface has been included so that users can compile libraries that can be loaded from the MScript interface and hook into the MScript parser to add commands and variables, and also add effective field calculators to ${\vec{H}}^{\text{eff}}$ not originally shipped with MERRILL. This should allow MERRILL to be adopted to a wide range of problems.

For viewing solutions, the authors recommend ParaView. It is a free, open source 3D viewer, easily downloaded and installed from the ParaView website, which can open and visualize solutions generated by MERRILL from the **WriteMagnetization** command. A plugin for ParaView for opening MERRILL solutions with some preprocessing already done can be found in the demo directory.

## Figures and Tables

**Figure A1:**
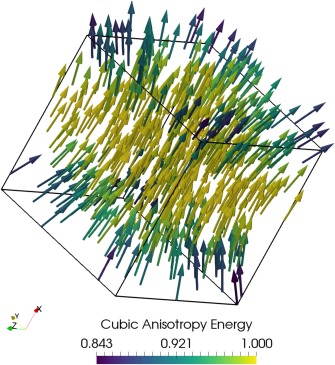
An initial flower solution at edge length $8.45\times l_{\text{exch}}$, colored by the cubic anisotropy energy.

**Figure A2:**
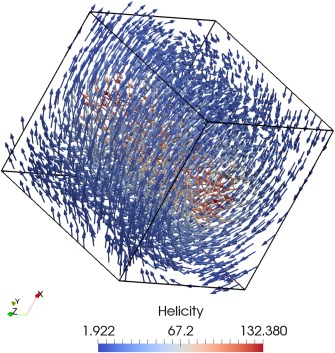
An initial vortex state at edge length $8.45\times l_{\text{exch}}$ colored by helicity ( $\vec{M}\cdot (\vec{\nabla }\times \vec{M})$).

**Figure A3:**
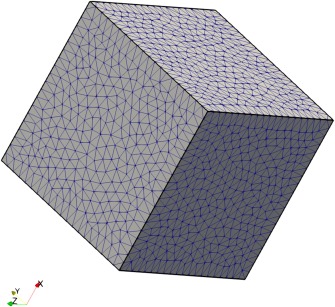
Cube mesh with a node spacing of 0.5 $\times l_{\text{exch}}$. Critical edge length versus node spacing.

**Figure A4:**
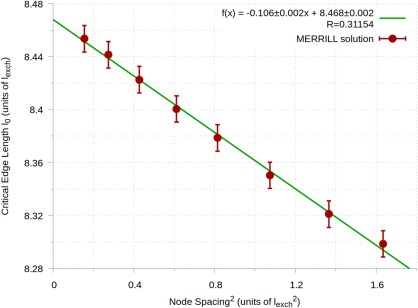
The critical length versus the node spacing of the mesh, and linear fit extrapolating the node spacing to 0. This shows the critical edge length for an infinitely fine mesh using MERRILL is $(8.468\pm 0.002)\times l_{\text{exch}}$. Critical energy versus node spacing.

**Figure A5:**
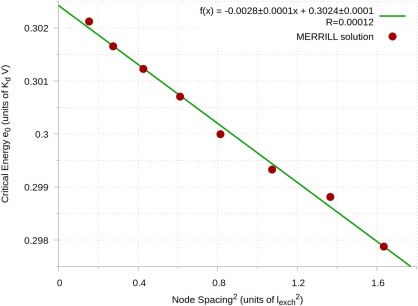
The critical energy versus the node spacing of the mesh, and linear extrapolating the node spacing to 0. This shows the critical energy for an infinitely fine mesh using MERRILL is $(0.30242\pm 0.00008)\times K_{d}V$.

**Figure C1:**
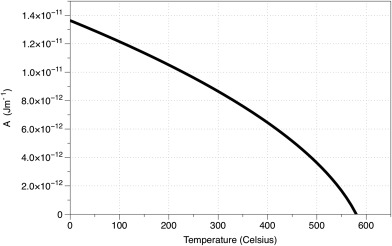
Exchange coupling versus temperature for magnetite.

**Figure C2:**
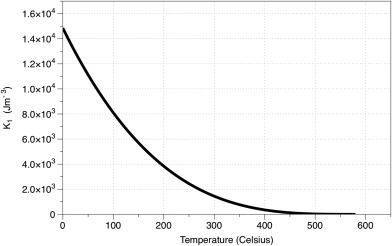
Crystalline anisotropy constant versus temperature for magnetite.

**Figure C3:**
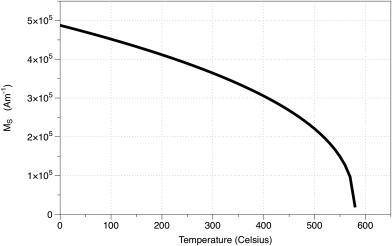
Saturation magnetization versus temperature for magnetite.

**Figure C4:**
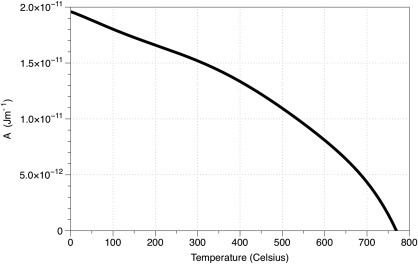
Exchange coupling versus temperature for iron.

**Figure C5:**
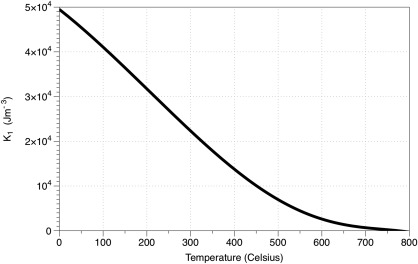
Crystalline anisotropy constant versus temperature for iron.

**Figure C6:**
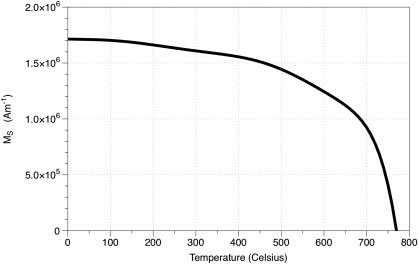
Saturation magnetization versus temperature for iron.

## Appendix A *μ*MAG Standard Problem 3

### A1. Introduction

The *μ*MAG Standard Problem 3 is a test for the critical edge length of a cube with uniaxial anisotropy for a change in the magnetic phase from a flower state to a single vortex state (http://www.ctcms.nist.gov/∼http://rdm/mumag.org.html). It was introduced in 1998.

In Standard Problem 3, the material parameters of the cube: the saturation magnetization *M_s_*, and the uniaxial anisotropy constant *K_u_*, are related by the relation:

(A1) $$K_{u}=0.1\frac{1}{2}\mu_{0}M_{s}^{2}$$

and the exchange length $l_{\text{exch}}$ is given in terms of the uniaxial anisotropy constant, the exchange coupling *A*

(A2) $$l_{\text{exch}}=\sqrt{\frac{A}{K_{u}}}.$$

Existing results suggest the critical edge length, where a flower state and a vortex state should have the same energy is in the region of 8.45 $\times l_{\text{exch}}$ to 8.55 $\times l_{\text{exch}}$.

### A2. Method

The material parameters used were

(A3) $$M_{s}=\;4.807680\times 10^{5}\;A/m,$$

(A4) $$A=\;1.334870\times 10^{-11}\;J/m,$$

(A5) $$K_{u}=\;1.452282\times 10^{4}\;{J/m}^{3}.$$

The exchange length was therefore

(A6) $$l_{\text{exch}}=9.587248\times 10^{-3}\;\mu m.$$

To nucleate a flower state, the mesh is scaled to 7.5 $\times l_{\text{exch}}$ and a local energy minimum (LEM) is found, then rescaled to 8.45 $\times l_{\text{exch}}$, our expected lower bound, and the LEM found and saved to disk. By “scaled to,” we mean the mesh is resized until edge length of the cube is the given value. This two step approach to nucleation is needed, since a flower state is not guaranteed to nucleate at $8.45\times l_{\text{exch}}$, as this is around the critical edge length; reminimizing at 8.45 $\times l_{\text{exch}}$ is done primarily to save time during minimization later.

For any particular domain state, its form will change slightly with grain size. A flower at a larger grain size, for example, will have a more divergent magnetization at the grain surface than a flower state at a smaller grain size. In that manner, states from grains closer in size tend to be more “similar” to each other than states from grains less close in size. Since the size 8.45 $\times l_{\text{exch}}$ is closer in size to our expected critical region than the initial size of 7.5 $\times l_{\text{exch}}$, the flower state at 8.45 $\times l_{\text{exch}}$ should be more “similar” to the states about the region of interest too. As a result, minimizing from the more “similar” state at 8.45 $\times l_{\text{exch}}$ to states about the region of interest takes less time than minimizing from the state at 7.5 $\times l_{\text{exch}}$. An initial flower state at 8.45 $\times l_{\text{exch}}$ is shown in Figure A1.

To nucleate a vortex state, the mesh is first scaled to 11 $\times l_{\text{exch}}$ to guarantee vortex nucleation and the LEM found, then scaled to 8.45 $\times l_{\text{exch}}$ and the LEM found and saved to disk. Again, reminimization saves a lot of time as the vortex at 8.5 $\times l_{\text{exch}}$ is more “similar” to the states around the region of interest than the state at 11 $\times l_{\text{exch}}$ is. An initial vortex state at 8.45 $\times l_{\text{exch}}$ is shown in Figure A2.

To evaluate, say, the flower state energy at a certain cube size, say *L*, the mesh is scaled to length *L*, the flower state at is loaded from disk, which for the flower was found for 8.45 $\times l_{\text{exch}}$, and the energy is minimized. The energy is then found and written to disk. This is done for the flower and vortex states for a range of cube sizes about the expected critical point of 8.5 $\times l_{\text{exch}}$.

The critical length for the cube is then found by finding the iteration just before the flower state energy passes the single vortex energy and the iteration just after, and using a linear interpolation between the energies of each iteration to find the edge length where they intercept. Denoting the first iteration's flower energy, vortex energy, and length scale as $f_{-},v_{-},l_{-}$, and the second iteration's as $f_{+},v_{+},l_{+}$, the intercept length scale, *l*_0_, is

(A7) $$l_{0}=l_{-}+\frac{\left(l_{+}-l_{-})(f_{-}-v_{-}\right)}{\left(f_{-}-v_{-})-(f_{+}-v_{+}\right)}.$$

This assumes the energies scale linearly with the edge length, which is incorrect, but for sufficiently small steps of the edge length, it should be accurate enough for this application.

This entire process is then repeated for an increasingly fine mesh. The critical edge length of the cube should scale as the square of the mesh spacing. This is due to higher resolution of the derivatives, which, for the demagnetization and exchange calculations are of the order two. By performing a linear fit on the (mesh spacing)^2^ versus the intercept length, it is be possible to extrapolate the intercept length for an infinitely fine mesh, i.e., where the mesh spacing is zero.

For each mesh, the energies were found for the flower and vortex states for 8.39 $\times l_{\text{exch}}$ to 4.70 $\times l_{\text{exch}}$ in steps of 0.01 $\times l_{\text{exch}}$. The mesh spacings used were 1.0 $\times l_{\text{exch}}$ to 0.3 $\times l_{\text{exch}}$ in steps of 0.1 $\times l_{\text{exch}}$. The meshes were generated using a Delaunay triangulation algorithm from the CGAL library (The CGAL Project, 2017). An example mesh at node spacing 0.5 $\times l_{\text{exch}}$ is given in Figure A3.

### A3. Results

From Figure A4, the critical edge length for an infinitely fine mesh was found to be $(8.468\pm 0.002)\times l_{\text{exch}}$, and from Figure A5, the critical energy was $(0.30242\pm 0.00008)\times K_{d}V$. A break down of the demag, exchange, and anisotropy energies at the critical length for the flower and vortex states is in Table A1. The average magnetization for these states at the critical length is in Table A2.

**Table A1:** *Extrapolated Partial Energies at the Critical Edge Length in Units of* $K_{d}V$

	Flower	Vortex
Demag	0.2791 ± 0.0001	0.0775 ± 0.0006
Anisotropy	0.00563 ± 0.00002	0.0520 ± 0.0001
Exchange	0.01773 ± 0.00005	0.1729 ± 0.0007

**Table A2:** *Extrapolated Magnetizations at the Critical Edge Length in Units of M_s_*

	Flower	Vortex
$〈m_{x}〉$	0.97086 ± 0.00008	−0.0000 ± 0.0008
$〈m_{y}〉$	−0.0000 ± 0.0002	0.0 ± 0.3
$〈m_{z}〉$	−0.0003 ± 0.0002	0.0000 ± 0.0007

These measurements were also carried out with MERRILL's built in regular mesh generation for cubes, and the difference in results was within 1%.

### A4. Discussion

MERRILL's solutions to the *μ*MAG Standard Problem 3 are in very good agreement with the other submissions. This is good evidence that the energy terms in MERRILL are correctly determined and converge to the true continuum value when mesh size approaches zero.

The error values presented here are for the extrapolation of the critical values MERRILL would find on an infinitely fine mesh. They are not errors in the actual values generated by MERRILL compared to the physical equivalent. There are many sources of error unaccounted for by this approach, including material parameters, mesh geometry, numerical errors, and approximation errors. For this reason, when comparing between MERRILL's results, and other submissions to the *μ*MAG site, it may be more informative to directly compare the extrapolated values than to compare the extrapolated values within the presented errors.

## Appendix B MERRILL Commands

The basic functionality of MERRILL provides a boundary element micromagnetic energy calculation for arbitrary particle shapes.

To use MERRILL requires at least

1. A particle geometry (e.g., a cube or octahedron) that describes the particle to be modeled. This geometry is translated into a tetrahedral mesh with a large set of model nodes by a mesh generator. The distance between the nodes defines the resolution of the model. Several ready to use meshes are provided together with MERRILL, but arbitrary meshes can be loaded as long as they fulfill some minimal quality criteria. Besides commercial software, there exist several professional open source mesh generators that are more than sufficient for generating simple particle meshes. For example, http://engits.eu/en/engrid, http://sourceforge.net/projects/netgen-mesher/, and http://geuz.org/gmsh/, http://meshlab.sourceforge.net/.
2. Material properties of the magnetic material to be modeled and the scaling constant determining the particle size. MERRILL provides a database of material properties for several common natural magnetic minerals.
3. With the above prerequisites it is already possible to do very sophisticated model runs resulting, e.g., in micromagnetic energies and mean magnetization values. To visualize the resulting output files MERRILL is set up to easily interact with ParaView.

### B1. Interpreter Language

MERRILL is operated through an interpreter language script that determines all aspects of the micromagnetic calculation.

The script is a simple ASCII file containing a sequence of lines. Empty lines, leading, trailing, and multiple spaces or tabs are ignored, as well as anything behind an exclamation mark (!). A number of keywords are used to call subroutines or perform simple assignments. All keywords are case insensitive, e.g., “ReadMesh” and “readmesh” are equivalent. The script file is parsed line by line. Each valid line is immediately interpreted and executed.

#### B1.1. Basic Commands

The following list contains the currently available commands.

***Set*** 〈 ***variable*** 〉 〈 ***value*** 〉 is used to define global variables for the material, the geometry of the mesh, or program parameters. The following *variables* are supported:

***Ms*** saturation magnetization in A/m.

***K1*** anisotropy constant for uniaxial or cubic anisotropy in J/m^3^.

***Aex*** exchange constant in J/m.

***Ls*** inverse length scale 1/m. Internally Ls^2^ is used.

***mu*** related to permeability of free space via mu $=\mu_{0}/4\pi$.

***NEBSpring*** Spring constant for nudged‐elastic band method (NEB).

***CurvatureWeight*** Weight of curvature contribution for nudged‐elastic band method (NEB).

***MaxMeshNumber*** Maximal number of finite element meshes stored. Must be set once before loading meshes.

***PathN*** Number of structures along the magnetization path. Warning: The mesh must have been defined previously! Use only after ReadMesh.

***ExchangeCalculator*** Chooses the exchange energy discretization method used. The available choices are 1= m Δm, 2= $\varphi^{2}$ along edges, 3= ${\left(\nabla \vartheta \right)}^{2}+\sin^{2}\vartheta \;{\left(\nabla \varphi \right)}^{2}$, 4= $\varphi^{2}$ from centroid to vertices.

***MaxRestarts*** Maximum number of restarts during energy minimization.

***MaxEnergyEvaluations*** Maximum number of energy calculations during energy minimization (typical: 5,000–10,000). Afterward energy minimization is aborted.

***MaxPathEvaluations*** Maximum number of path energy calculations during path minimization (typical: 500–2,000).

***Zone*** Current Zone to be written into the TecPlot output file (double). Zone can be set before each output, or c an be used with automatic increment.

***ZoneIncrement*** Automatic increment of zone (default = 1.0).

***AllExchange*** Values greater zero imply that all exchange energy calculations are performed and logged. This slows the program down.

***Magnetite*** 〈 ***temperature*** 〉 **C** Defines material constants for magnetite at temperature 〈 *temperature* 〉 to be used in subsequent calculations. The temperature is given in degrees Celsius and must be positive and below magnetite's Curie temperature at 580°C.

***Resize*** 〈 ***old length*** 〉 〈 ***new length*** 〉 changes the length scale of the mesh such that after this rescaling the length 〈 *old length* 〉 will become 〈 *new length* 〉.

***(Cubic*** | ***Uniaxial) Anisotropy*** Defines the symmetry of the anisotropy energy.

***CubicRotation*** 〈φ,ϑ,α〉 Rotates the cubic easy axes using the rotation matrix with the prescribed Euler angles.

***CubicAxes*** $〈a_{x},a_{y},a_{z}〉\;〈b_{x},b_{y},b_{z}〉\;〈c_{x},c_{y},c_{z}〉$ Directly set the 3 cubic axes, $\vec{a},\;\vec{b}$, and $\vec{c}$, for the cubic anisotropy. Users should ensure the axes are mutually orthogonal.

***EasyAxis*** 〈x,y,z〉 Determines the easy axis for uniaxial anisotropy.

***External field direction*** 〈x,y,z〉 Determines the direction vector of an external homogenous magnetic field. Intrinsically sets the field to *B* = 1 T.

***External field strength*** 〈 ***B*** 〉 〈 ***unit*** 〉 Determines the strength of the external homogenous magnetic field as 〈 *B* 〉 in units of 〈 *unit* 〉. Possible values for 〈 *unit* 〉 are “μT,” “mT,” or “T.” Must be set after defining the direction. Subsequent calls reset the field to 〈B 〉 without changing the direction. Can be used for hysteresis modeling.

***ReadMesh*** 〈 ***index*** 〉 〈 ***filename*** 〉 Reads the Patran file 〈 *filename* 〉, and stores the corresponding mesh and finite element arrays at location 〈 *index* 〉 *. The index must be less or equal to the previously set MaxMeshNumber*.

***LoadMesh*** 〈 ***index*** 〉 Loads a previously read mesh and its finite element arrays from location 〈 *index* 〉. This mesh will then be used in subsequent operations.

***ReadMagnetization*** 〈 ***filename*** 〉 Reads a magnetization file, 〈 *filename* 〉, (.dat) into the current mesh magnetization array. Make sure that it was created for the currently active mesh! The magnetization read is used in subsequent operations.

***Uniform magnetization*** 〈x,y,z〉, [〈b〉] Creates a uniform magnetization for the current mesh pointing in the normalized direction 〈x,y,z〉. The optional parameter 〈b〉 is the index of the block of nodes in the mesh that should be set. By definition block 1 contains the free nodes, while higher block numbers can be used to define fixed nodes. These blocks have to be defined in the Patran file. Any previous magnetization is lost.

***Vortex magnetization*** $〈x,y,z〉,\;[〈x_{0},y_{0},z_{0}〉],\;[〈v〉],\;[$ **RH** | **LH** ] Creates an approximate vortex state with core pointing along the line from ${\vec{x}}_{0}$ to $\vec{x}$, with component 〈v〉 along the core direction, and right handed (RH), or left handed (LH) orientation. By default, ${\vec{x}}_{0}=\vec{0}$, *v* = 0 and the orientation is RH.

***Invert magnetization*** $〈s_{x},s_{y},s_{z}〉$ Inverts the current magnetization structure by multiplying each component of $\left(m_{x},m_{y},m_{z}\right)$ with the corresponding sign $\left(s_{x},s_{y},s_{z}\right)$. For example, Invert magnetization 1 −1 1 on each node changes the current magnetization $\left(m_{x},m_{y},m_{z})\mapsto (m_{x},-m_{y},m_{z}\right)$.

***Randomize magnetization*** 〈 ***angle*** 〉 Randomly changes each current magnetization vector by at most 〈 *angle* 〉 degrees. The previous magnetization is (partially) lost.

***Randomize all moments*** Replaces the current magnetization by randomly distributed unit vectors. Any previous magnetization is lost!

***ReMesh*** 〈 ***index*** 〉 Takes the current magnetization array and interpolates it at the nodes of the previously read mesh at location 〈*index*〉. This mesh from location 〈*index*〉 is then loaded and will be used in subsequent operations.

***ConjugateGradient*** Uses conjugate gradient steps during the accelerated descent.

***SteepestDescent*** Uses normal gradient steps during the accelerated descent.

***Minimize*** Calls the minimization routine for the current mesh and initial magnetization. This call does not save the final result!

***EnergyLog*** 〈 ***filename*** 〉 Starts logging all subsequent energy calculations into the logfile 〈 *filename* 〉.log. Logging can be stopped by *EndLog* or *CloseLogfile*

***CloseLogfile*** ends the previous logging of energy calculations or path minimizations.

***WriteMagnetization*** 〈 ***filename*** 〉 Saves the magnetization and the mesh in two files:

〈 *filename* 〉.dat contains vertex coordinates and magnetization vectors.

〈 *filename* 〉_ *mult.dat* TecPlot file for visualization using ParaView or TecPlot. Contains mesh geometry and one or more magnetization states.

***WriteHyst*** 〈 ***filename*** 〉 Saves hysteresis data in 5 columns of ${\vec{H}}^{\text{zm}}\cdot \vec{M},\;|{\vec{H}}^{\text{zm}}|$ and the 3 components on the average unit magnetization vector, $〈m_{x}〉,\;〈m_{y}〉,\;〈m_{z}〉$, where M is the magnetization and $\left|H^{\text{zm}}\right|$ is the magnitude of external field. $\vec{M}\cdot {\vec{H}}^{\text{zm}}$ is normalized to the saturated magnetization in the direction of the applied field.

***SystemCommand*** 〈 ***command*** 〉 … Performs the system command in the remaining arguments as a line. No guarantee can be given for correct behavior. Uses FORTRAN's *SYSTEM* command.

***KeyPause*** Pauses script evaluation and waits for Key + Enter for continuation.

***GradientTest*** Test feature to check energy gradient calculation against several finite‐difference estimates.

***(Stop*** | ***End)*** Stops script evaluation.

#### B1.2. Path Related Commands

The following contains all path related commands available for NEB calculations.

***MagnetizationToPath*** 〈 ***index*** 〉 Saves the current magnetization in the path at location 〈 *index* 〉. This allows to assemble a path from individual magnetization states that have to fit to the current mesh! After assembling a path it must be renewed before further operations can be performed.

***PathToMagnetization*** 〈 ***index*** 〉 Moves the path magnetization state at location 〈 *index* 〉 to the current magnetization. This allows to change individual magnetizations in the path. For example, Initial and final states of a path read from a file can be minimized for new material constants.

***RenewPath*** Defines all path variables, like distances and tangent vectors, assuming that all magnetizations have been correctly filled.

***RefinePathTo*** 〈 ***newlength*** 〉 Refines the current path to a new number of states by linear interpolation in the magnetization angles. This also resets *PathN* to the new value and renews the path. Of course, the new number of states can also be less than the previous *PathN*.

***WriteTecPlotPath*** 〈 ***filename*** 〉 Exports the current path to a TecPlot file with name 〈 *filename* 〉. All states along the path are individual zones in the TecPlotFile.

***ReadTecPlotPath*** 〈 ***filename*** 〉 Reads a new path from a TecPlot file with name 〈 *filename* 〉. All states along the path are individual zones in the TecPlotFile. Because this also reads in the mesh, all mesh related quantities are recalculated. Sets *PathN* and allocates necessary space for mesh and path arrays.

Make sure that all material parameters are correctly assigned, since those are not read!

***MakeInitialPath*** Assumes that a path is defined by *set PathN* 〈 *number* 〉 and that the first and last magnetization patterns are defined. Then proceeds by stepwise minimization to construct an initial path for subsequent optimization by the NEB method.

***PathMinimize*** Assumes that an initial path is defined and minimizes the action integral using a variant of the NEB method.

***PathLogfile*** 〈 ***filename*** 〉 Starts logging all subsequent path minimization calculations into three logfiles 〈 *filename* 〉.enlog 〈 *filename* 〉.grlog, and 〈 *filename* 〉.dlog. They contain energies along the path, norms of the gradients along the path and cumulative distances along the path. Logging can be stopped by *EndLog* or *CloseLogfile*.

***PathStructureEnergies*** [〈 ***filname*** 〉] Calculates the energies for each structure along the currently loaded path (in Joule). If 〈 *filename* 〉 is omitted, the path is written to standard output.

***Energy*** Calculates the energy (in Joule) of the currently loaded magnetization structure.

#### B1.3. Interpreter Language Control Constructs

The following commands are simple options of the interpreter language to implement loops or script variables. Note that they cannot be nested or used recursively.

***Loop*** 〈 ***variable*** 〉 〈 ***startvalue*** 〉 〈 ***endvalue*** 〉 [〈 ***step*** 〉] Takes all commands until the next *EndLoop* statement and performs a loop over the enclosed commands by replacing the variable 〈 *variable* 〉 with values from 〈 *startvalue* 〉 to 〈 *endvalue* 〉 in steps of 〈 *step* 〉. If step is not given step‐size *step = 1.0* is assumed. Within the loop the string #〈variable〉 is replaced by the integer value of *variable*, the string %〈variable〉 is replaced by the double precision value of *variable*, and the string $〈variable〉$ is replaced by a string of the value of *variable*. Nested loops are not yet supported. Warning: The *Loop* command itself must NOT contain other variables than the loop variable. This is so because currently the parsing for replacing variables is performed only after unravelling the loops.

***EndLoop*** Delimits the set of commands inside the active loop.

***Define*** 〈 ***variable*** 〉 〈 ***value*** 〉 Defines a numeric variable that can be used like a loop variable.

***AddTo*** 〈 ***variable*** 〉 〈 ***value*** 〉 Adds a number to a previously defined variable.

***Undefine*** 〈 ***variable*** 〉 Forgets a previously defined variable.

## Appendix C Material Parameters

The micromagnetic models require values of the three temperature‐dependent magnetic parameters of saturation magnetization (*M_s_*), the crystalline anisotropy constant (*K*_1_), and the exchange constant (*A*). Each parameter is represented by a polynomial, based on the best fit to experimental data.

### C1. Magnetite

For magnetite the experimental data was derived from Heider and Williams (1988) for the exchange constant, Fletcher and O'Reilly (1974) for the crystalline anisotropy and Heider et al. (1987) for the saturation magnetization. The polynomial expressions used in MERRILL for each of these three magnetite parameters is

(C1) $$A(T)=\frac{\sqrt{21622.526+816.476\times (T_{C}-T)}-147.046}{408.238\times 10^{11}},$$

(C2) $$K_{1}(T)=-2.13074\times 10^{-5}\times {\left(T_{C}-T\right)}^{3.2},$$

(C3) $$M_{s}(T)=737.384\times 51.876\times {\left(T_{C}-T\right)}^{0.4},$$

where *T* is the temperature in Celsius and *T_C_* is the Curie temperature for magnetite taken as 580 C. These parameter polynomials are plotted in Figures C1, C2, C3.

### C2. Iron

For iron the experimental data for *M_s_* is derived from Crangle and Goodman (1971) and Cullity and Graham (2008), where the sparsity of data near the Curie temperature in tabulated values of Crangle and Goodman (1971) are supplemented data digitized from the *M_s_*‐*T* graphs of Cullity and Graham (2008). For *K*_1_, the experimental data are taken from Honda et al. (1928) reproduced in graphical form in Cullity and Graham (2008). Although this *K*_1_ material parameters fall to zero as the temperature approached the Curie point, more recent studies (e.g., Muxworthy & Williams, 2015) use a slightly higher room temperature value. In order to reconcile this discrepancy, the data from Honda et al. (1928) was scaled by a factor (480/456). Finally the exchange constant, *A*, for iron was obtained following the method outlined in Heider and Williams (1988), using the stiffness constant values reported by Stringfellow (1968).

The polynomial expressions used in MERRILL for each of these there iron parameters is

(C4) $$A(T)=\begin{array}{llll} & -1.8952\times 10^{-12} & & +3.0657\times 10^{-13}\;T\\ & -1.599\times 10^{-15}\;T^{2} & & +4.0151\times 10^{-18}\;T^{3}\\ & -5.3728\times 10^{-21}\;T^{4} & & +3.6501\times 10^{-24}\;T^{5}\\ & -9.9515\times 10^{-28}\;T^{6} & & \end{array},$$

(C5) $$M_{s}(T)=\begin{array}{llll} & +1.75221\times 10^{6} & & -1.21716\times 10^{3}\;T\\ & +33.3368\;T^{2} & & -0.363228\;T^{3}\\ & +1.96713\times 10^{-3}\;T^{4} & & -5.98015\times 10^{-6}\;T^{5}\\ & +1.06587\times 10^{-8}\;T^{6} & & -1.1048\times 10^{-11}\;T^{7}\\ & +6.16143\times 10^{-15}\;T^{8} & & -1.42904\times 10^{-18}\;T^{9} \end{array},$$

(C6) $$K_{1}(T)=\begin{array}{llll} & (+54967.1 & & +44.2946\;T\\ & -0.426485\;T^{2} & & +0.000811152\;T^{3}\\ & -1.07579\times 10^{-6}\;T^{4} & & +8.83207\times 10^{10}\;T^{5}\\ & -2.90947\times 10^{-13}\;T^{6})\times \frac{480.0}{456.0} & & \end{array}.$$

Note for iron, *T* is the temperature in Kelvin, although the default temperature unit in MERRILL is Celsius. The conversion to Kelvin is done internally. These parameter polynomials are plotted in Figures C4, C5, C6.
